# Assessing the Pathogenic Ability of *Ralstonia pseudosolanacearum* (*Ralstonia solanacearum* Phylotype I) from Ornamental *Rosa* spp. Plants

**DOI:** 10.3389/fpls.2017.01895

**Published:** 2017-11-02

**Authors:** Napoleon N. A. Tjou-Tam-Sin, Jeroen L. J. van de Bilt, Marcel Westenberg, Peggy P. M. A. Gorkink-Smits, N. Marco Landman, Maria Bergsma-Vlami

**Affiliations:** National Reference Centre, Dutch National Plant Protection Organization (NPPO), Wageningen, Netherlands

**Keywords:** *Ralstonia pseudosolanacearum*, *Rosa* spp., disease severity, disease incidence, temperature dependency, stem inoculation, soil drenching

## Abstract

*Ralstonia pseudosolanacearum* (*Ralstonia solanacearum* phylotype I) isolates found in stunted, yellowing, and wilted ornamental rose (*Rosa* spp.) were assessed for their pathogenic ability in two rose cultivars (cv. “Armando” and cv. “Red Naomi”) and in four solanaceous crops: tomato (*Solanum lycopersicum* cv. “Money Maker”), tobacco (*Nicotiana tabacum* cv. “White Burley”), eggplant (*Solanum melongena* cv. “Black Beauty”) and sweet pepper (*Capsicum annum* cv. “Yolo Wonder”). Significant differences were observed in susceptibility between the two rose cultivars as well as between the two modes of inoculation performed. The cultivar “Armando” was significantly more susceptible than cultivar “Red Naomi,” exhibiting higher disease severity and incidence. Similarly, stem inoculation after wounding was found to be significantly more effective than soil drenching, resulting in higher disease severity. Additionally, a temperature dependency in susceptibility was observed for both cultivars irrespective of the mode of inoculation, however, this was significantly more pronounced upon soil drenching. The solanaceous crops all showed to be susceptible to the *R*. *pseudosolanacearum* isolates originated from the *Rosa* spp. plants. Furthermore, both rose cultivars were able to harbor symptomless infections with other *R*. *pseudosolanacearum* and *R. solanacearum* isolates than those isolated from rose. Our results clearly demonstrated that latent infections in a rose cultivar such as cv. “Red Naomi” do occur even at temperatures as low as 20°C. This latency poses high risks for the entire floricultural industry as latently infected *Rosa* spp. plants are propagated and distributed over various continents, including areas where climatic conditions are optimal for the pathogen.

## Introduction

*Ralstonia solanacearum* (*R*. *solanacearum*) has been recently classified as the second most important plant pathogenic bacterium, in terms of scientific and economic relevance (Mansfield et al., 2012). Its significance is to be attributed to its persistence, systemic nature, wide host range, broad geographical distribution, and huge genome plasticity (Elphinstone, 2005; Denny, 2006; Castillo and Greenberg, 2007). More specifically, *R. solanacearum* genes involved in virulence evolve at a much faster rate than its genome as a whole (Remenant et al., 2010). The species *R*. *solanacearum*, as positioned taxonomically by Yabuuchi et al. (1995) appeared to be a heterogeneous species exhibiting considerable variation in virulence to diverse hosts (Prior and Steva, 1990; Marin and El-Nashaar, 1993; Jaunet and Wang, 1999). It was additionally proven to be a species complex (Palleroni and Doudoroff, 1971; Roberts et al., 1990; Gillings et al., 1993; Taghavi et al., 1996) that might represent more than one true species (Fegan and Prior, 2005). The complete group of strains belonging to this species complex is referred to as the “*R. solanacearum* species complex” (RSSC) (Fegan and Prior, 2005). Strains of the RSSC are traditionally divided into five races based on host range (Buddenhagen et al., 1962; He et al., 1983), and six biovars based on utilization of three disaccharides and three hexose alcohols (Hayward, 1964, 1994a). With the development of molecular tools for bacterial characterization, the classification of the RSSC has undergone major changes. As a result, a hierarchical classification system based on phylogenetic relationships was introduced by Fegan and Prior (2005). The four defined phylotypes showed to be correlated with the geographical origin of the strains. Phylotype I correlates with strains originating from Asia, phylotype II with those from America, phylotype III with those from Africa, and phylotype IV with those from Indonesia, Australia and Japan, including the strains of *Ralstonia syzygii*. More recently, a revision of the RSSC was proposed (Safni et al., 2014), introducing the division of the “species complex” into three genospecies: *R. solanacearum, Ralstonia pseudosolanacearum* and *R. syzygii*. The genospecies *R. solanacearum* consists of strains of *R. solanacearum* phylotype II only, including the type strain. *R. pseudosolanacearum* is composed of *R. solanacearum* strains that belong to phylotypes I and III. Finally, *R. syzygii* contains only *R. solanacearum* phylotype IV strains.

Although the RSSC has been studied intensively for decades, still new host species have been recently reported (Chandrashekara and Prasannakumar, 2010; Prieto Romo et al., 2012; Lin et al., 2015; Jiang et al., 2016; Tjou-Tam-Sin et al., 2017). The potential movement of RSSC representatives via propagative material between countries has probably been underestimated. The risk of bacterial dissemination might even be greater in cases where plants are considered non-hosts. When new undescribed RSSC pathotypes are introduced via propagative material into a new environment, the potential impact on local crops in this new environment is greatly unpredictable (Norman et al., 2009b). The increased trade in plant products around the world together with the rapid adaptation potential of RSSC (Genin and Boucher, 2004) can result in major outbreaks of this disease on known and on new host plants. Recently, isolates of *R. pseudosolanacearum* (phylotype I, race 1, biovar 3) were isolated from ornamental roses showing typical bacterial wilt symptoms and were confirmed to cause the disease symptoms (Tjou-Tam-Sin et al., 2017). Objective of this study was to assess the virulence of *R. pseudosolanacearum* isolates (phylotype I, race 1, biovar 3) acquired from symptomatic rose plants. The pathogenic ability of these isolates on two commercial rose cultivars, as well as on tomato, tobacco, eggplant and sweet pepper plants was investigated. Additionally, the pathogenic ability of a panel of *R. pseudosolanacearum* and *R. solanacearum* isolates originating from plants other than rose and belonging to phylotype I, II, and III has been evaluated on rose. The influence of temperature, botanical variation between rose cultivars and mode of inoculation (stem inoculation after wounding versus soil drenching) on susceptibility and disease conductivity was additionally evaluated.

## Materials and methods

### Bacterial isolates and culture conditions

Eight representative *R. pseudosolanacearum* and *R. solanacearum* isolates selected according to their phylotype, geographic origin, and host of origin (Table 1) were assessed for their virulence on *Rosa spp*. and on *Solanaceae*. Isolates PD 7123, PD 7195, and PD 7216 were isolated in 2015 from naturally-infected *Rosa* spp., exhibiting typical symptoms and originating from glasshouse production systems in different locations in the Netherlands (Tjou-Tam-Sin et al., 2017). More specifically, PD 7123 was isolated from cv. “Red Naomi,” PD 7195 from cv. “Maritiem,” and PD 7216 from cv. “Armando.” Bacteria were isolated on YPG agar medium or on SMSA after incubation at 28°C for 2 or 6 days, respectively. Bacterial cultures were stored lyophilised as well as in sterile water at room temperature. Phylotype determination assigned the PD 7123, PD 7195, and PD 7216 isolates to phylotype I (Fegan and Prior, 2005).

**Table 1:** *Ralstonia solanacearum* and *Ralstonia pseudosolanacearum* strains used in this study.

**Strain**	**Collection ID**	*Ralstonia* species^*^	**Phylotype**	**Biovar**	**Race**	**Host of origin**	**Geographic origin**
PD 1945	LMG 2297	*R*. *pseudosolanacearum*	I	3		*Casuarina equisetifolia*	Republic of Mauritius
PD 7123		*R*. *pseudosolanacearum*	I	3	1	*Rosa* spp.	The Netherlands
PD 7216		*R*. *pseudosolanacearum*	I	3	1	*Rosa* spp.	The Netherlands
PD 7195		*R*. *pseudosolanacearum*	I	3	1	*Rosa* spp.	The Netherlands
PD 4500		*R*. *solanacearum*	II	1		*Begonia* spp.	Costa Rica
PD 2762		*R*. *solanacearum*	II	2	3	*Solanum tuberosum*	The Netherlands
PD 7221	NCPPB 325	*R*. *solanacearum*	II	1		*Solanum lycopersicum*	USA
PD 1940	LMG 9673	*R*. *pseudosolanacearum*	III	1		*Pelargonium capitatum*	Réunion (France)

* *According to Safni et al. (2014)*.

### Pathogenicity assays

Two independent experiments were performed.

#### Design experiment 1

In Experiment 1, eight *R. pseudosolanacearum* and *R. solanacearum* isolates were included for the inoculations (Table 1) in two rose cultivars (cv. “Armando” and cv. “Red Naomi”) and in four solanaceous crops: tomato (*Solanum lycopersicum* cv. “Money Maker”), tobacco (*Nicotiana tabacum* cv. “White Burley”), eggplant (*Solanum melongena* cv. “Black Beauty”), and sweet pepper (*Capsicum annum* cv. “Yolo Wonder”). The number of replicates per combination of plant species × isolate was: *n* = 3 for each of the rose cultivars inoculated and *n* = 10 for each of the inoculated solanaceous plant species. Suspensions of the bacterial isolates were prepared from 48 to 72 h old cultures by suspending them in phosphate buffer (PB 0.01M) to a final concentration of ca 10^8^ CFU/mL. Sterile phosphate buffer (PB 0.01M) has been used as negative control. Rose plants were directly inoculated into the stem after pre-injuring their woody stems by wounding them with a scalpel. Thereafter, 20–50 μl bacterial suspension was injected per plant with a syringe and needle (21G × 2″; 0.8 × 50 mm) into the stem wounds (stem inoculation after wounding). For the four solanaceous crops, stem inoculations were performed by injuring them by stabbing with the needle (25G × 5/8″; 0.6 × 16 mm) of a syringe and leaving a drop of bacterial suspension behind on both entrances of the wound. Each stem was inoculated at two sites: at its lower part, just above the soil level and a few centimeters higher. Inoculated plants were kept at a 24/21°C diurnal temperature regime for up to 42 days post inoculation (dpi). Monitoring of disease was performed by assessing the disease incidence up to 42 dpi. Disease incidence was scored as the percentage (%) of symptomatic plants in a plant x isolate treatment, at 42 dpi, relatively to the total number of plants included in that treatment.

#### Design experiment 2

**Figure 1:**
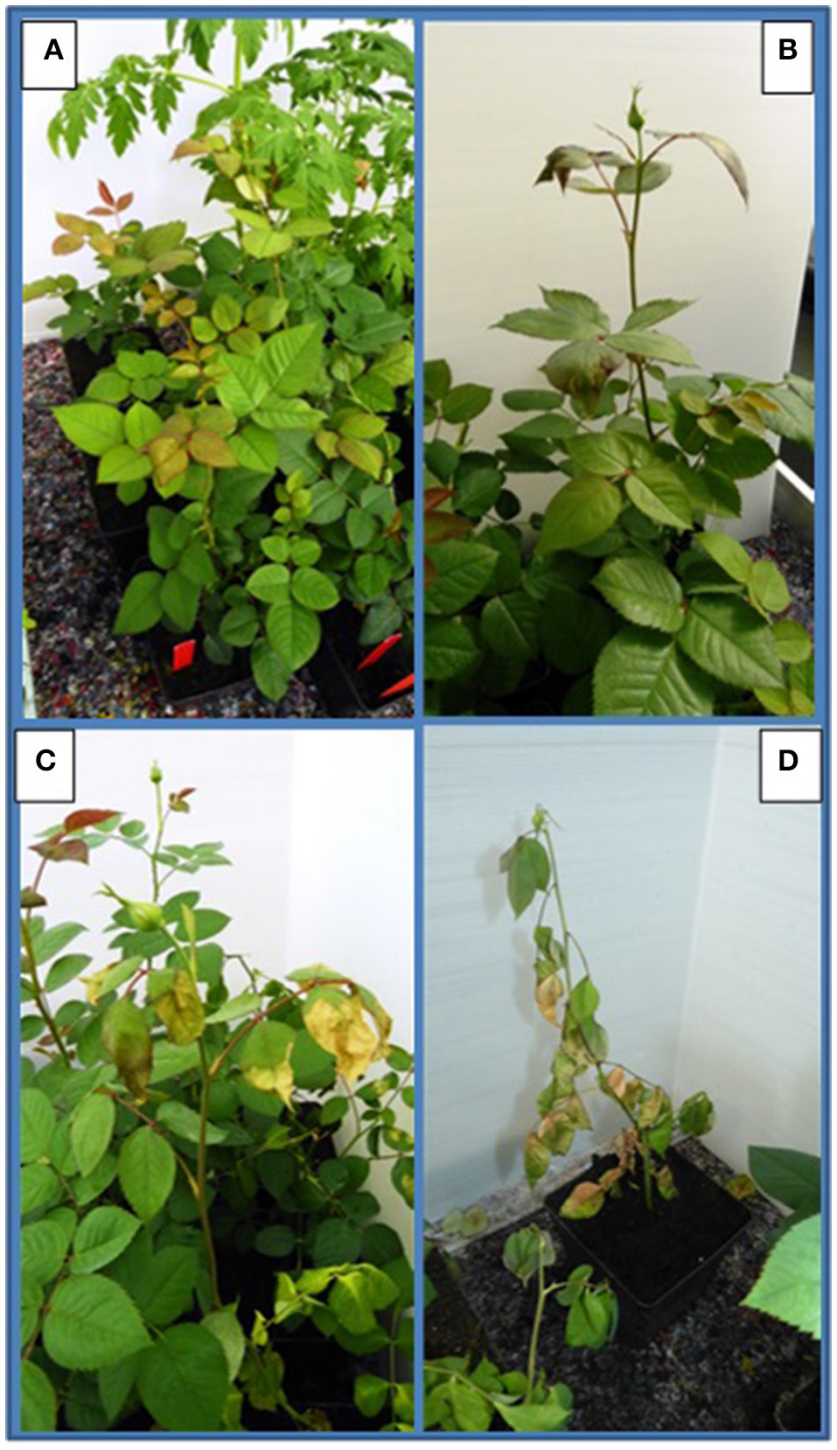
Assessment of disease severity by making use of a scale categorizing the symptoms induced by *Ralstonia pseudosolanacearum* observed on *Rosa* spp. according to level 0–3 (corresponding with pictures **A–D**): **(A)** (0 = no symptoms), **(B)** (1 = starting symptoms), **(C)** (2 = clear, typical symptoms), **(D)** (3 = plant death).

In experiment 2, two *R. pseudosolanacearum* (PD 7123 and PD 7216) isolates (Table 1) were inoculated in *Rosa* sp. cv. “Armando” and cv. “Red Naomi”, and in tomato (*S. lycopersicum* cv. “Money Maker”). Experiment 2 was executed at two temperature levels (20 and 28°C) and inoculations were performed either by 1. Stem inoculation after wounding or by 2. Soil drenching. Preparation of bacterial suspensions and stem inoculation after wounding were both performed as described in Experiment 1. However, a final bacterial concentration of ca 10^7^ CFU/mL was used for both, stem inoculation after wounding and soil drenching. For soil drenching, a total volume of 100 mL of bacterial suspension was poured onto the rooted soil of a plant potted in peaty soil (volume per pot, ca. 1.5 liter). Sterile phosphate buffer (PB 0.01M) has been used as negative control. The number of replicates per combination of plant species × isolate × temperature × mode of infection was: *n* = 10 for inoculations directly into the stem after wounding and *n* = 12 for soil drenching. Plants were kept at 20°C and at 28°C for up to 116 dpi. Disease progress was monitored initially at four dpi, thereafter, for the first 2 weeks, at a frequency of twice up to three times per week and thereafter once per week. At each observation point, disease severity was assessed only for the plant (per treatment) that was in the most advanced disease stage (Figure 1). At the end of experiment 2 (at 116 dpi), the disease incidence (%) and the disease severity were assessed for the total number of plants included.

Disease severity was assessed by using a scale as follows: 0 = healthy plants; 1 = first appearance of doubtful or mild symptoms of bacterial wilt; 2 = typical bacterial wilt expression with rolling and hanging leaves, leaf necrosis protruding from the leaf margins toward the leave veins, yellowing of leaves and early leaf drop, and possibly dark brown to black necrosis of stems dying back; 3 = irreversible total wilt and plant death, later followed by dark brown necrosis of the stems (Figure 1).

Observation data were analyzed in order to assess whether the isolate, the temperature, the botanical variation and the mode of inoculation significantly influenced disease severity. Analysis of variance (ANOVA) was performed using SPSS version 17.0. Statistical significance was accepted at α = 0.01.

### Latency testing and identity confirmation of re-isolates

In both experiments, re-isolations on SMSA medium were performed from at least one individual plant per treatment where typical symptoms were developed, in order to confirm the *R. pseudosolanacearum* and *R. solanacearum* infections. The identity of re-isolates exhibiting typical colony morphology for *R. pseudosolanacearum* and *R. solanacearum* on SMSA medium was confirmed by TaqMan PCR (Weller et al., 2000) with a minor grove binding unit (mgb)-adjusted probe as described in Vreeburg et al. (2016). Plants showing no symptoms till the end of the experiments were also included for the re-isolations. For this, extract from a ca. 2 cm section stem tissue per each stalk of a rose plant (avoiding the point of inoculation and at least 1 cm stem jointed to the inoculation point) was plated on SMSA medium, in order to confirm either the absence of *R. pseudosolanacearum* and *R. solanacearum* infections or the presence of latent infections. For experiment 1, all plants within one treatment combination were pooled for re-isolation at 42 dpi. For experiment 2, re-isolation was performed from each plant individually at 116 dpi.

## Results

### Experiment 1

Disease incidence (%) in *Rosa* spp. and in the four solanaceous plant species at 42 dpi when incubated at a 24°C, is presented in Table 2. Both, *Rosa* sp. cultivars and the four solanaceous plant species were highly susceptible for the isolates PD 7123, PD 7216, and PD 7195 originating from the naturally-infected rose. Disease incidence in both *Rosa* sp. cv. “Armando” and *Rosa* sp. cv. “Red Naomi” was 100% at 42 dpi, with plants exhibiting typical disease symptoms. Plant death at 42 dpi was observed only for cv. “Armando” plants. However, cv “Red Naomi” plants developed typical symptoms for *R. pseudosoalanacearum* in rose as in Figure 1C, but they did not exhibit plant death within the 42 dpi period. Remarkably, upon stem inoculation after wounding with a panel of *R. pseudosolanacearum* and *R. solanacearum* isolates belonging to phylotypes I, II, and III (Table 1) both cv. “Armando” and cv. “Red Naomi” were able to harbor infections, however, without showing symptoms at 42 dpi (Table 2). These symptomless infections on rose plants at 24°C were confirmed by the results obtained from latency testing.

**Table 2:** Disease incidence (%) of bacterial wilt on two ornamental rose cultivars (*n* = 3) and four solanaceous plant species (*n* = 10) after stem inoculation with eight *Ralstonia solanacearum* and *Ralstonia pseudosolanacearum* isolates at 24/21°C diurnal temperature regime, 42 dpi, (Experiment 1).

**Isolate**	**Original host**	**Disease incidence (%)**					
		***Rosa* sp. cv. Armando**	***Rosa* sp. cv. Red Naomi**	***Solanum lycopersicum***	***Nicotiana tabacum***	***Solanum melongena***	***Capsicum annuum***
PD 1945	*Casuarina equisetifolia*	0^*^	0^*^	80	20	100	30
PD 7123	*Rosa spp*.	100	100	100	90	100	100
PD 7216	*Rosa spp*.	100	100	100	90	100	90
PD 7195	*Rosa spp*.	100	100	100	90	100	100
PD 4500	*Begonia* spp.	0^*^	0^*^	50	70	100	^**^nd
PD 2762	*Solanum tuberosum*	0^*^	0^*^	100	^**^nd	100	^**^nd
PD 7221	*Solanum lycopersicum*	0^*^	0^*^	100	50	100	100
PD 1940	*Pelargonium capitatum*	0^*^	0^*^	30	10	70	^**^nd

* *Latently infected*.

** *nd = not done*.

Irrespective of the *R. pseudosolanacearum* (phylotype I) isolate (PD 7123, PD 7216, and PD 7195) inoculated on cv. “Armando”, the first symptomatic plant at 24°C appeared at 6–7 dpi, with isolate PD 7123 being slightly delayed in symptom development. However, the first symptomatic cv. “Red Naomi” plant appeared later; at 11 dpi (for PD 7216), at 13 dpi (for PD 7195), and at 15 dpi (for PD 7123). On the other hand, irrespective of the *R. pseudosolanacearum* (phylotype I) isolate (PD 7123, PD 7216, and PD 7195) used for inoculation, the first plant exhibiting plant death was observed at 21 dpi, only for *Rosa* sp. cv. “Armando”. From *Rosa* sp. cv. “Red Naomi” no plants exhibited plant death within 42 dpi. These results underline the difference in susceptibility for bacterial wilt caused by *R. pseudosolanacearum* (phylotype I) between rose cultivars.

Disease incidence in all four solanaceous plant species inoculated at 24°C with the *R. pseudosolanacearum* (phylotype I) isolates PD 7123, PD 7216, and PD 7195 was 90–100% at 42 dpi (Table 2). Irrespective of the isolate (PD 7123, PD 7216, and PD 7195) used for inoculation and irrespective of the solanaceous plant species used, the first symptomatic solanaceous plant always appeared within 4 dpi. However, the first plant exhibiting plant death in all four solanaceous plant species has been monitored between 6 and 21 dpi: up to 6–11 dpi for tomato, eggplant, and sweet pepper, and 21 days for tobacco.

### Experiment 2

**Figure 2:**
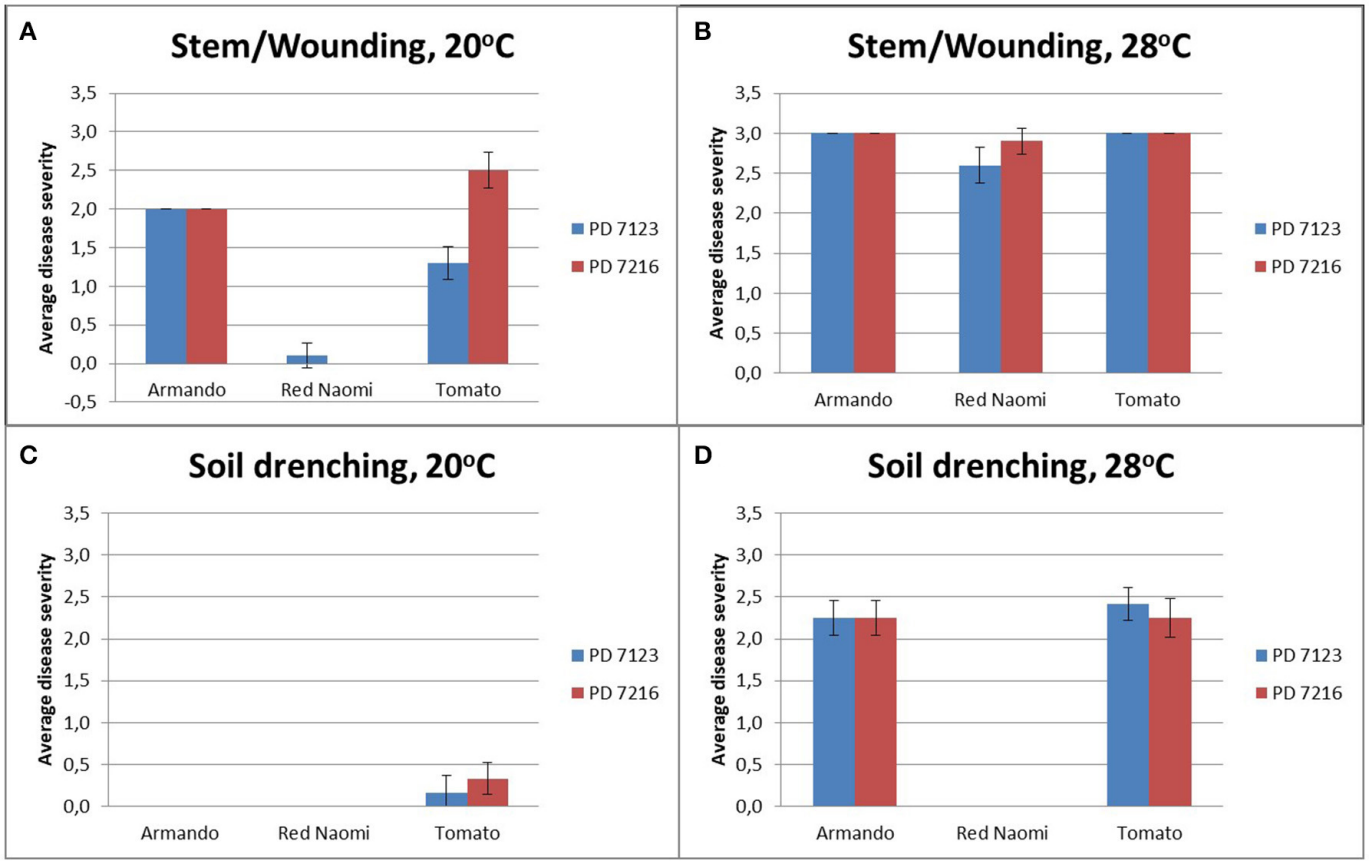
Average disease severity of bacterial wilt caused by *Ralstonia pseudosolanacearum* isolates PD 7123 and PD 7216 on plants of *Rosa* sp. cv. “Armando,” *Rosa* sp. cv. “Red Naomi” and *Solanum lycopersicon*, cv. “Money Maker,” at 20°C **(A,C)** and 28°C **(B,D)** at 116 dpi. Disease severity is given as the average of *n* = 10 plants for the stem inoculation after wounding **(A,B)** and *n* = 12 plants for the soil drenching treatment **(C,D)**. Error bars indicate the standard error observed.

There were no significant differences (*P* = 0.078) in disease severity between the isolates PD 7123 and PD 7216 (Table 3). However, significant differences in disease severity have been indicated by ANOVA for the other main factors: temperature, mode of inoculation and botanical variation (Table 3). With the exception of the “temperature^*^botanical variation^*^mode of inoculation” interaction (*P* < 0.0005), all other interactions were not significantly different (Table 3). Disease severity was in general lower in treatment combinations including cv. “Red Naomi,” at 20°C, and after soil drenching application (Figure 2). However, it is worthwhile to mention that although cv. “Armando” plants produced typical symptoms upon stem inoculation after wounding at 20°C, they did not exhibit plant death within 116 dpi. Additionally, only one cv. “Red Naomi” plant, inoculated with isolate PD 7123, showed mild symptoms within 116 dpi upon stem inoculation after wounding at 20°C. Re-isolations from the symptomless cv. “Red Naomi” plants within the treatment combination of stem inoculation after wounding at 20°C revealed high levels of latent infections, namely 60 and 70% for isolates PD 7123 and PD 7216, respectively (Table 4).

**Table 3:** ANOVA analysis of fixed effects and interactions on severity of bacterial wilt caused by *Ralstonia pseudosolanacearum* upon stem inoculation after wounding (*n* = 10) or soil dreching (*n* = 12), at 116 dpi (Experiment 2).

**Effect^a^**	**df**	**Error df**	***F*-value**	***P*-value**
Inoc	1	240	3.127	0.078
Temp	1	240	347.333	<0.0005^#^
BV	2	240	87.003	<0.0005^#^
MI	1	240	257.673	<0.0005^#^
Temp^*^Inoc	1	240	0.666	0.415
Temp^*^BV	2	240	1.125	0.326
Temp^*^MI	1	240	0.907	0.342
Inoc^*^BV	2	240	2.170	0.116
Inoc^*^MI	1	240	1.184	0.278
BV^*^MI	2	240	0.882	0.415
Temp^*^Inoc^*^BV	2	240	2.165	0.117
Temp^*^Inoc^*^MI	1	240	0.666	0.415
Temp^*^BV^*^MI	2	240	60.876	<0.0005^#^
Inoc^*^BV^*^MI	2	240	0.643	0.527
Temp^*^Inoc^*^BV^*^MI	2	240	2.165	0.117

a *Inoc, Inocula (isolates PD 7123 and PD 7216); Temp, Temperature (20°C and 28°C); BV, Botanical variation (Rosa sp. cv. Armando, Rosa sp. cv. Red Naomi, and tomato cv. Money Maker); MI, Mode of inoculation (stem inoculation after wounding and soil drenching)*.

# *significant at α = 0.01*.

**Table 4:** Disease incidence (%) of bacterial wilt caused by *Ralstonia pseudosolanacearum* isolates PD 7123 and PD 7216 at 20 and 28°C on *Rosa* sp. cv. Armando, *Rosa* sp. cv. Red Naomi, and *Solanum lycopersicum*, cv. Money Maker, upon stem inoculation after wounding (*n* = 10) or soil drenching (*n* = 12), at 116 dpi (Experiment 2).

**Temperature**	**Inoculation method**	**Disease incidence (%)**					
		***Rosa*** **sp. cv. Armando**		***Rosa*** **sp. cv. Red Naomi**		***Solanum lycopersicum***	
		**PD 7123**	**PD 7216**	**PD 7123**	**PD 7216**	**PD 7123**	**PD 7216**
20°C	Stem inoculation	100	100	10/(60)^*^	0/(70)^*^	50	100
20°C	Soil drenching	0	0	0	0	8/(17)^*^	17/(17)^*^
28°C	Stem inoculation	100	100	90	100	100	100
28°C	Soil drenching	75	75	0	0	92	83

* *% incidence/(% latently infected)*.

**Figure 3:**
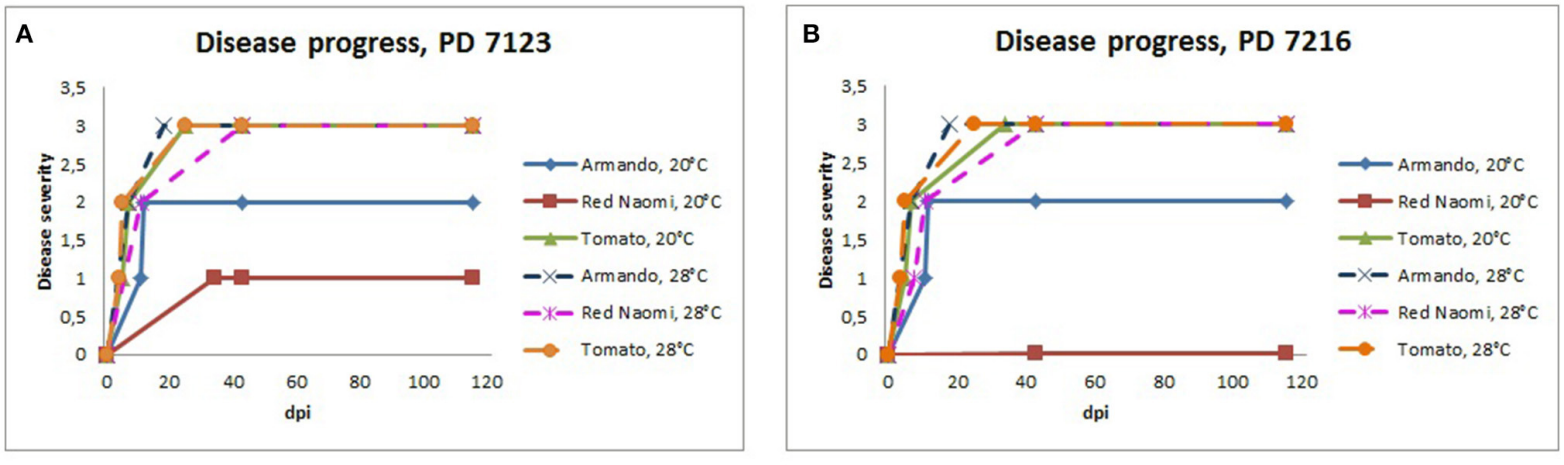
Severity of bacterial wilt caused by *Ralstonia pseudosolanacearum* isolates PD 7123 **(A)** and PD 7216 **(B)** on plants of *Rosa* sp. cv. “Armando,” *Rosa* sp. cv. “Red Naomi” and *Solanum lycopersicum*, cv. “Money Maker” in time, at 20 and 28°C up to 116 dpi. At each observation day, an assessment was done of disease severity of one plant (per treatment) that was in the most advanced disease stage.

Stem inoculation after wounding in both cv. “Armando” and cv. “Red Naomi” at 28°C showed typical symptoms and exhibited plant death. The first plant exhibiting plant death at 28°C has been monitored already after 18 dpi for cv. “Armando” and after 43 dpi for cv. “Red Naomi” (Figure 3). These observations acquired by stem inoculation after wounding underline a statistically significant effect due to the interaction between the cultivar, the temperature and the mode of inoculation (Table 3), that is also supported by the disease progress curves (Figure 3). No significant differences (*P* = 0.116) were observed between cv. “Armando” and cv. “Red Naomi” upon inoculation with either isolate PD 7123 or PD 7216 in terms of disease severity (Table 3).

The effect of temperature was statistically significant (*P* < 0.0005) after application of soil drenching in cv. “Armando” but also in *S. lycopersicum* cv. “Money Maker” plants (Table 3). After soil drenching at 20°C, both cv. “Armando” and cv. “Money Maker” presented no symptoms and only minor disease expression [<20% disease incidence (Table 4) and average severity < 0.5% (Figure 2C)]. However, after soil drenching at 28°C higher values for disease incidence (Table 4) and disease severity (Figure 2D) were observed in both cv. “Armando” and cv. “Money Maker.” This effect on the disease severity was statistically supported (Table 3). Additionally, most cv. “Armando” plants exhibited plant death within the 116 dpi period of monitoring after soil drenching at 28°C. On the other hand, cv. “Red Naomi” plants showed no symptoms at all after soil drenching, irrespective of the incubation temperature, for up to 116 dpi (Figures 2C,D). Testing of the symptomless cv. “Red Naomi” plants after soil drenching at 28°C revealed no latent infections (Table 4). This highlights the need of mechanical injury to result in an assessable amount of disease severity (Figure 2) and disease incidence (Table 4) in cv. “Red Naomi” at either 20°C or 28°C and in cv. “Armando” at 20°C. Within the symptomless *S. lycopersicum* cv. “Money Maker” plants, upon soil drenching, re-isolations after 116 dpi at 20°C revealed the presence of latent infections at a level of 17% (Table 4). In the treatment combination of stem inoculation after wounding at 20°C, our results indicated that the disease severity (Figures 2A, 3) and disease incidence (Table 4) in both, cv “Armando” and *S. lycopersicum* cv. “Money Maker” were higher than those observed in cv “Red Naomi.”

## Discussion

Bacterial wilt of ornamental and crop field plants caused by RSSC is frequently catastrophic, resulting in major economic losses in many countries (Hayward, 1991). Although, traditionally the most predominantly represented plant families harboring hosts of *R. pseudosolanacearum* and *R*. *solanacearum* are the *Solanaceae* and *Musaceae* (Buddenhagen, 1961; Hayward, 1994b; Elphinstone, 2005; Alvarez et al., 2010; Albuquerque et al., 2014), still new host species have been recently reported belonging to different plant families (Chandrashekara and Prasannakumar, 2010; Prieto Romo et al., 2012; Lin et al., 2015; Jiang et al., 2016). Recently, we reported, for the first time worldwide, the finding of *R. pseudosolanacearum* (phylotype I, race 1, biovar 3), causing bacterial wilt in *Rosa* spp. plants under greenhouse cultivation in the Netherlands (Tjou-Tam-Sin et al., 2017). The main aim of our study was to assess the virulence of *R. pseudosolanacearum* isolates from naturally-infected rose plants on two representative commercial cultivars of *Rosa* spp., namely cv. “Armando” and cv. “Red Naomi.”

Since disease development in plants only occurs when a virulent pathogen, a susceptible host and a favorable environment are simultaneously present, the influence of a number of abiotic parameters have been evaluated in our study; i.e., the of influence temperature and mode of inoculation (stem inoculation after wounding or soil drenching). Significant differences in disease severity among the 20°C and the 28°C treatments were found, irrespective of the mode of inoculation, with disease severity and disease incidence being consistently higher at the 28°C treatments. As strains of *R*. *pseudosolanacearum* (phylotype I, race 1, biovar 3) are generally known to require a relatively high optimum temperature (24–35°C) for symptom development (Kelman, 1953; Bocsanczy et al., 2014), the outcome of our study showing high disease expression at 28°C but only poor to moderate at 20°C is in accordance with the expectation. An additional explanation for the relatively poor disease development at 20°C, may be a virulence barrier that RSSC representatives exhibit under low temperature conditions, at the stage of host rhizosphere colonization (Bocsanczy et al., 2012).

In general, stem inoculation after wounding resulted in higher disease severity and disease incidence than soil drenching at both temperatures, however, this was more pronounced at 20°C than at 28°C. Up to 116 dpi, cv. “Red Naomi” showed no symptoms upon stem inoculation after wounding at 20°C, however, re-isolations resulted in high levels of latent infections (60–70%). This implicates a high risk for import of propagative plant material potentially harboring latent infections when kept at temperature as low as 20°C. The effectiveness of soil drenching on susceptibility to bacterial wilt showed to be significantly low for cv. “Red Naomi,” irrespective of the temperature, as no symptoms were monitored upon soil drenching for up to 116 dpi. However, no latent infections were found in this case, underlining a high dependency of mechanical stem injury in the infection process for effective disease development in cv. “Red Naomi.” On the other hand, highly susceptible cultivars such as cv. “Armando” can be effectively infected upon soil drenching via the root system. Based on our data, the use of stalks acquired from rose cut flowers as source of propagation material in nurseries poses a potentially high risk for the dissemination of *R. pseudosolanacearum* through systemic (latently) infected *Rosa* sp. plants and especially under greenhouse cultivation conditions, as previously highlighted (Norman and Yuen, 1998, 1999) for propagative plant material, in general.

The influence of botanical variation between cv. “Armando” and cv. “Red Naomi” on susceptibility to bacterial wilt is statistically supported in our experiments. This is in accordance with previous studies performed on other plant species (Jaunet and Wang, 1999; Norman et al., 2009a; Lebeau et al., 2011). On the other hand, no differences in virulence were observed among the isolates PD 7123, PD 7216, and PD 7195, irrespective of the temperature, mode of inoculation or botanical variation. These isolates are genetically closely related and formed a distinct monophyletic clade in phylotype I (*unpublished data*). Remarkably, both cv. “Armando” and cv. “Red Naomi” upon stem inoculation after wounding by a panel of RSSC isolates belonging to phylotypes I, II, and III were able to harbor latent infections at 42 dpi. Among these RSSC isolates inoculated in rose, an isolate of *R. solanacearum* phylotype IIB was included, formerly referred to as race 3 biovar 2 which is adapted to temperate climates and is known as the causal agent of potato brown rot. Our observations of harbored latent infections of this and other RSSC isolates in rose implicates an additional risk for import of latently infected rose plants by other RSSC isolates than the one isolated from rose. Our results highlight the urgency to study these latent RSSC infections upon exposure to different environmental conditions (i.e. temperature, water stress) and beyond the 6 week period (42 dpi).

The present work confirmed that *R. pseudosolanacearum* isolates PD 7123, PD 7216, and PD 7195 acquired from naturally-infected rose can cause severe disease to young plants of Solanaceae plants. The disease incidence within the Solanaceae plants was 90–100% at 42 dpi upon stem inoculation after wounding at 24°C. However, the susceptibility of potato to *R. pseudosolanacearum* (phylotype I) isolates from rose was not included in this study, as previously done for *R. solanacearum* phylotype II isolates from pelargonium (Williamson et al., 2002). In the current study we mainly concentrated on the risk of the *R. pseudosolanacearum* (phylotype I) isolates from rose toward glasshouse cultivated crops in temperate climatic regions. However, in warmer climatic zones *R. pseudosolanacearum* biovar 1 isolates are known to infect outdoor potato crops (Cruz et al., 2008). Our results highlight the urgency to study the pathogenic ability of *R. pseudosolanacearum* (phylotype I) isolates from rose on potato as well.

In conclusion, this study showed that temperature, botanical variation and mode of inoculation, alone or in combination, can significantly influence disease severity in rose upon inoculation with *R*. *pseudosolanacearum* (phylotype I, race 1, biovar 3) isolates from naturally-infected rose plants in greenhouse cultivation in the Netherlands. Additionally, it underlined the ability of these isolates to occur frequently as latent infections in the plant. This represents a serious challenge for the entire floricultural industry, worldwide. Although our results greatly contribute to the knowledge of the epidemiology of the *R. pseudosolanacearum* from rose, yet the limited host plants included here illustrate that the precise host range of this pathogen is still poorly characterized and greatly unknown.

## Author contributions

Conceived and designed the experiments: NT, MB. Performed the experiments NT, JvdB, PG, ML, MW. Analyzed the data: NT. Wrote the paper: NT, MB.

### Conflict of interest statement

The authors declare that the research was conducted in the absence of any commercial or financial relationships that could be construed as a potential conflict of interest.
